# Overexpression of p16^INK4a^ in Urothelial Carcinoma In Situ Is a Marker for MAPK-Mediated Epithelial-Mesenchymal Transition but Is Not Related to Human Papillomavirus Infection

**DOI:** 10.1371/journal.pone.0065189

**Published:** 2013-05-28

**Authors:** Julie Steinestel, Marcus V. Cronauer, Johannes Müller, Andreas Al Ghazal, Peter Skowronek, Annette Arndt, Klaus Kraft, Mark Schrader, Andres J. Schrader, Konrad Steinestel

**Affiliations:** 1 Department of Urology, University of Ulm, Ulm, Germany; 2 Gemeinschaftspraxis for Pathology Augsburg, Augsburg, Germany; 3 Institute of Pathology and Molecular Pathology, Bundeswehrkrankenhaus Ulm, Ulm, Germany; 4 Bundeswehr Institute of Radiobiology, Munich, Germany; 5 Institute of Pathology, University of Ulm, Ulm, Germany; University of Colorado, United States of America

## Abstract

**Background:**

The role of human papillomavirus (HPV) in bladder carcinogenesis remains controversial. Overexpression of p16^INK4a^, a surrogate marker for infection with oncogenic HPV in other tumours, has been described for urothelial carcinoma *in situ* (UCIS). Our goal was therefore to evaluate whether overexpression of p16^INK4a^ is associated with HPV infection and to identify mechanisms of p16^INK4a^ upregulation in UCIS.

**Materials and Methods:**

In 60 tissue specimens from a total of 45 patients (UCIS and controls), we performed p16^INK4a^ immunohistochemistry followed by detection and subclassification of HPV DNA. In a subset of samples, we tested for gene amplification of p16^INK4a^ applying fluorescence *in situ* hybridization (FISH). RAS/MAPK signalling and epithelial-mesenchymal transition (EMT) was assessed using immunohistochemistry. Finally, we transfected urothelial carcinoma cells with *KRAS* and examined the expression of p16^INK4a^ as well as markers of EMT.

**Results:**

We found overexpression of p16^INK4a^ in 92.6% of UCIS and in all cervical intraepithelial neoplasia (CIN) controls. In contrast, we detected high-risk HPV DNA in 80% of CIN, but none in UCIS. There was no gene amplification of p16^INK4a^. High levels of phosphorylated kinases and urokinase plasminogen activator (uPA) and loss of membraneous E-cadherin were detected in UCIS. *KRAS* transfection of urothelial carcinoma cells led to upregulation of p16^INK4a^ and uPA accompanied by loss of E-cadherin that could be inhibited by application of the kinase-inhibitor Sorafenib.

**Conclusions:**

Our results show that overexpression of p16^INK4a^ in UCIS is neither associated with HPV infection nor p16^INK4a^ gene amplification but is a consequence of enhanced RAS/MAPK signalling that promotes EMT, possibly due to Sorafenib-sensitive paracrine secretion of the EMT activator uPA. These findings might open a novel therapeutic option for localized but aggressive urothelial cancer.

## Introduction

Urothelial bladder cancer is the 7^th^ most common cancer in men and the 17^th^ most common cancer in women worldwide[1]. Besides genetic predisposition, known risk factors for the disease include smoking and chronic exposure to aromatic amines and polycyclic aromatic hydrocarbons[1]. Urothelial carcinoma *in situ* (pTis, UCIS) has a high potential to progress to invasive urothelial carcinoma[2]. In animal models, it has been shown that UCIS lesions can be induced by exposure to the carcinogene nitrosamine and that they progress to invasive carcinomas upon STAT3 activation, *P53* mutations and loss of heterozygosity of chromosome 9[3], [4]. It has further been shown that UCIS lesions already express molecular markers that conform to epithelial-mesenchymal transition (EMT) of the tumour cells and are regarded as a prerequisite for invasion and metastasis[5]. Unfortunately, the diagnosis of UCIS is sometimes challenging. For urologists, it can be hard to distinguish flat lesions from reactive bladder wall during urocystoscopy[6], [7]. In histology, reactive urothelial atypia (UA) and UCIS share common characteristics, such as loss of nuclear stratification and an increase in mitotic activity[8]. Therefore, different immunohistochemical and genetic markers have been previously tested to aid in the differential diagnosis[9]. One of the most promising markers for confirmation of the diagnosis of UCIS is p16^INK4a^, a protein that physiologically acts as a tumour suppressor and inductor of cellular senescence[10]. Yin and colleagues were able to show that UCIS displays strong immunoreactivity for p16^INK4a^ while UA did not[11]. However, the exact mechanism of p16^INK4a^ upregulation in UCIS is still unclear. Cervical and head and neck cancers display overexpression of the protein upon oncogenic infection with high-risk genotypes of human papillomavirus (HPV). Viral oncoprotein E7 interacts with p16^INK4a^ target RB, leading to its dissociation from the transcription factor E2F which then initiates transition from G to S phase of the cell cycle[12], [13]. Viral protein E6 induces inactivation of tumour suppressor p53. The subsequent upregulation of p16^INK4a^ protein expression, which is regarded as a surrogate marker for high-risk HPV infection in these tumours, is understood as an “unsuccessful attempt” to stop cell proliferation. However, it is still unclear whether high-risk HPV infection also promotes urothelial tumourigenesis[14]–[16]. Therefore, our attempt was to investigate whether the strong expression of p16^INK4a^ in UCIS is associated with HPV infection and, if this is not the case, to reveal the mechanism of p16^INK4a^ upregulation in these lesions.

## Materials and Methods

### Ethics statement

All tissue samples were collected for histologic examination and diagnostic purposes and were thoroughly anonymized for the use in this study. Thus no informed consent was needed. This study was approved by the University of Ulm ethics committee (Approval No. 104/2012 - May 10^th^, 2012).

### Tissue samples

45 patients and 60 tissue samples were included in the study. All samples were submitted to the Institute of Pathology, Bundeswehrkrankenhaus Ulm, or to the Gemeinschaftspraxis for Pathology, Augsburg, from January 1^st^, 2001 to December 31^st^, 2011. Clinico-pathological characteristics are summarized in Table 1.

**Table 1:** Clinico-pathological sample characteristics.

	Urothelium (%)	UCIS (%)	CIN (%)
Total patients (n = 45)	21	19	5
Age (yrs; m±SD)	74±10.48	74±9.93	31±7.39
m∶f	3∶18	17∶2	0∶5
Tissue samples (n = 60)	28	27	5
Inflammation	5(17.6%)	2(7.4%)	0(0%)
Adjacent inv. Ca	0	7(25.9%)	0(0%)
p16 Immunoreactivity	0(0%)	25(92.6%)	5(100%)
HPV DNA	n.a.	0(0%)	4(80%)

### Immunohistochemistry, image acquisition and expression analysis

Immunohistochemistry was done according to standard protocols as previously described [17] on a BenchMark Autostainer (Ventana Medical Systems, Tucson, USA); antibodies/working concentrations are listed in Table S1. Microscopic slide evaluation/image acquisition was performed using a Leica DM6000B light microscope (Leica, Wetzlar, Germany) and the *Diskus Mikroskopische Diskussion* image acquisition software (Carl H. Hilgers, Königswinter, Germany). P16^INK4a^ was regarded as positive if there was a diffusely distributed, strong cytoplasmic and nuclear staining signal.

### Human papillomavirus (HPV) testing

Whole genomic DNA was extracted from paraffin slides using the automated Maxwell® 16 FFPE Plus LEV DNA Purification Kit (Promega, Madison, USA). Amplification of HPV DNA with biotinylated primers and hybridization of the PCR products to strips precoated with specific oligonucleotide probes was performed using the HPV typing kit from AID diagnostics (Strassberg, Germany) according to the manufacturer's protocol. The kit detects HPV genotypes 6, 11, 16, 18, 31, 33, 35, 39, 45, 51, 52, 53, 56, 58 and 59.

### FISH analysis and statistics

FISH analysis was performed on five representative cases out of the 25 p16^INK4a^-positive UCIS. 5 µm-thick slides were cut off paraffin blocks and pretreated using standard procedures. Slides were incubated with the Vysis LSI *CDKN2A* SpectrumOrange/CEP 9 SpectrumGreen DNA probe (Abbott, Abbott Park, USA) and mounted in DAPI/Antifade-Solution (ZytoVision, Bremerhaven, Germany). The *CDKN2A* locus encodes for p16^INK4a^, while centromere 9 (CEP 9) was used as an internal positive control. Gene copy numbers and centromere 9 signals in each 100 nuclei of 5 UCIS lesions and of the underlying stroma were counted out and each nucleus was assigned to one of the following groups according to the number of visible *CDKN2A* SpectrumOrange DNA probe signals: 1 orange/2 green (1O2G), 2 orange/2 green (2O2G) or 3 or more orange/2 green (3+O/2G). The 3+O/2G pattern would therefore indicate gene amplification of the *CDKN2A* locus encoding for p16^INK4a^.

### Cell culture, transfection experiments and Western Blot

RT112 human urothelial carcinoma cells were authenticated and proven to be free of contamination with animal cells in August, 2012, by the Leibniz Institute/DSMZ (Braunschweig, Germany)(Fig. S2). *KRAS* mutation status was tested with *KRAS* strip assays (Vienna Labs, Vienna, Austria) as previously described (Fig. S3)[17]. Transfection was performed using the Optifect reagent (Invitrogen, Karlsruhe, Germany) according to the manufacturer's protocol. TNFα (SignalChem, Richmond, Canada) was added in 2 doses of each 10 ng 24 and 12 hours prior to cell lysis. Sorafenib (LKT Laboratories, St. Paul, USA) was dissolved in Dimethyl Sulfoxide (DMSO) and applied to a concentration of 5 µM 24 hours prior to cell lysis. Immunoblot was done according to standard methods (loading control: β-Actin; Antibodies/working concentrations listed in Table S1). Quantification after generation of lane profile plots was done using a software-based Gel Analyzer (ImageJ software, v. 1.46r, NIH, Bethesda, USA). Overexpression of KRAS was proven by RAS immunoblotting; insertion of mutated *KRAS* was proven by *KRAS* strip assay testing (Fig. S3)[17].

### Expression vectors

PcDNA3-KRAS-wild type/pcDNA3-KRAS-G12D (GGT/GAT transition) mammalian expression vectors were kind gifts of Dr. Patrizio Castagnola, National Cancer Research Center, Genova, Italy, and have been previously published[18].

### Immunofluorescence (IF) analysis

IF staining was performed according to standard methods as previously described by our work group[19]. Antibodies/working concentrations are listed in Table S1. Slides were mounted in vectashield aqueous mount containing 4,6-diamidino-2-phenylindole (DAPI)(Vector Laboratories, Burlingame, USA).

## Results

### Clinico-pathological data

The study contained 27 UCIS samples from 19 patients who had a mean age of 74 years (range, 50–86 years; Table 1). The male to female ratio was 17∶2. Two patients (7.4%) had underlying inflammation and seven patients (25.9%) suffered from invasive urothelial carcinoma at another localization of the bladder in addition to UCIS. The five female controls with cervical intraepithelial neoplasia (CIN III) had a mean age of 31 years (range, 24–44 years), and there was no adjacent inflammation or invasive carcinoma in these specimens. 21 patients with internal and external controls (normal urothelium/epithelium adjacent to UCIS/CIN III and tumour-free urothelial tissue samples) had a mean age of 74 years (range, 24–86 years). Of these, five urothelial controls (17.6%) presented with erosive urocystitis.

### Immunohistochemical analysis of UCIS and CIN

**Figure 1:**
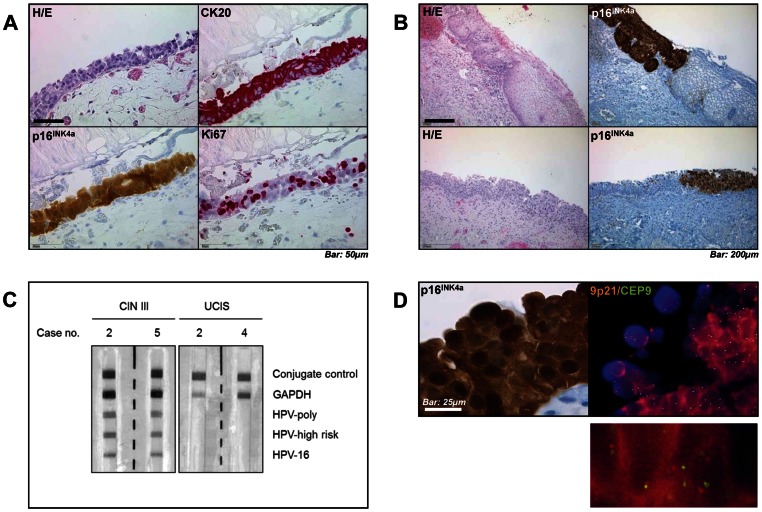
Immunohistochemical characterization, HPV testing and FISH analysis in UCIS. **A**, Histologic microphotograph of urothelial carcinoma in situ (UCIS) shows loss of nuclear stratification, positivity for Cytokeratin 20 and p16^INK4a^ as well as increased proliferative activity as indicated by Ki-67 immunostaining. **B**, Comparison between high-grade cervical intraepithelial neoplasia (CIN III, upper row) and UCIS (lower row) shows a comparable distribution of p16^INK4a^ immunopositivity. **C**, Reverse hybridization blotting detects high-risk human papillomavirus (HPV) genotype 16 DNA in representative CIN III, but not in UCIS samples. **D**, Fluorescence-in-situ-hybridization (FISH) analysis shows no amplification of *CDKN2A* gene encoding for p16^INK4a^ (orange) compared to control (centromere 9, green) in representative p16^INK4a^-positive UCIS. *Scale bars as indicated.*

UCIS was confirmed by immunohistochemistry for Cytokeratin 20 and the proliferation marker Ki67 in all included specimens (Figure 1A). Further analysis revealed strong nuclear and cytoplasmic positivity for p16^INK4a^ in 25/27 UCIS (92.6%) and 5/5 CIN lesions (100%) (Figure 1A and B, Table 1). Healthy and inflamed urothelium were negative for p16^INK4a^, and there was sharp demarcation between p16^INK4a^-positive UCIS/CIN and adjacent urothelium/epithelium (Figure 1B). There was no detectable p16^INK4a^ expression in inflamed urothelium (0/5, 0%; Figure S1A).

### Human papillomavirus (HPV) detection in UCIS and CIN

High-risk human papillomavirus (HPV) DNA (genotype 16) could be detected in 4/5 CIN lesions (80%; Table 1 and Figure 1C). For UCIS, there was no detectable HPV DNA in any of the specimens (0/27, 0%)

### Fluorescence in situ hybridization (FISH) analysis in UCIS

FlSH analysis highlighting the copy numbers of the *CDKN2A* gene locus encoding for p16^INK4a^(orange) and centromere 9 (control; green) per nucleus in 5 representative samples out of the 25 p16^INK4a^-positive UCIS specimens revealed two orange and two green signals (2O2G pattern) in 393/500 UCIS nuclei (78.6%; Figure 1D, Table 2). 107/500 UCIS nuclei (21.4%) showed loss of one *CDKN2A* orange signal (1O2G pattern). In the underlying stroma, 454/500 nuclei (90.8%) showed a 2O2G and 46/500 (9.2%) showed a 1O2G pattern. We did not detect three or more orange signals (3+O2G pattern) in any of the examined nuclei. These results show that there was no amplification, but rather loss of the *CDKN2A* gene locus encoding for p16^INK4a^ in neoplastic urothelium.

**Table 2:** Results of *CDKN2A/centromere 9* Fluorescence in situ (FISH) analysis.

	1O2G(%)	2O2G(%)	3+O2G(%)
UCIS (n = 500 nuclei)	107/500 (21.4%)	393/500 (78.6%)	0/500
Stroma (n = 500 nuclei)	46/500 (9.2%)	454/500 (90.8%)	0/500

### Immunohistochemistry for RAS/MAPK signaling activity and epithelial-mesenchymal transition in UCIS

**Figure 2:**
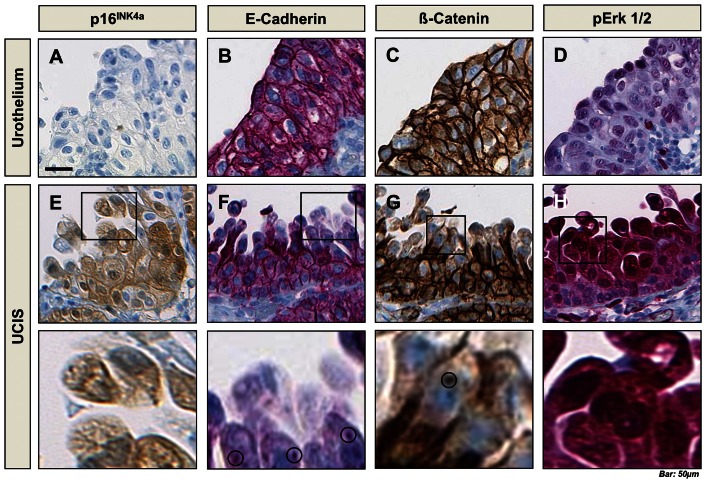
Further immunohistochemical characterization of normal urothelium and UCIS. **A-D**, Healthy urothelium shows negativity for p16^INK4a^, membraneous immunopositivity for E-cadherin and Beta-catenin and sparse positivity for phosphorylated ERK 1/2. **E-G**, UCIS shows cytoplasmic and nuclear positivity for p16^INK4a^ and loss of membraneous expression of E-cadherin and Beta-catenin. This is inversely correlated with strong cytoplasmic and nuclear positivity for pERK 1/2 in UCIS (**H**). Greater magnification shows intracytoplasmatic clusters of E-cadherin and nuclear clusters of Beta-catenin (**lower row**). *Scale bar: 50 µm*.

Immunohistochemical analysis showed no signal for p16^INK4a^ and weak nuclear staining for pERK1/2, but strong membraneous positivity for E-cadherin and Beta-catenin in healthy urothelium (Figure 2A-D). There was no staining signal for pAKT in UCIS and controls (Figure S1B). In UCIS, there was strong nuclear and cytoplasmic positivity for p16^INK4a^, while staining for E-cadherin and Beta-catenin was weaker in apical, discohesive tumour cells (Figure 2E-G). We found punctate staining pattern for E-cadherin in the cytoplasm and for Beta-catenin in the nucleus (bottom panel: higher magnifications from E-H). Loss of membraneous E-cadherin and Beta-catenin expression from basal to apical tumour cells was inversely associated with strong, cytoplasmic and nuclear positivity for phosphorylated ERK1/2 (Figure 2H). This is consistent with increased RAS/MAPK signaling activity and epithelial-mesenchymal transition (EMT) of the neoplastic, p16^INK4a^-positive urothelium.

### p16^INK4a^ expression and epithelial-mesenchymal transition in transfected and sorafenib-treated cell lines

**Figure 3:**
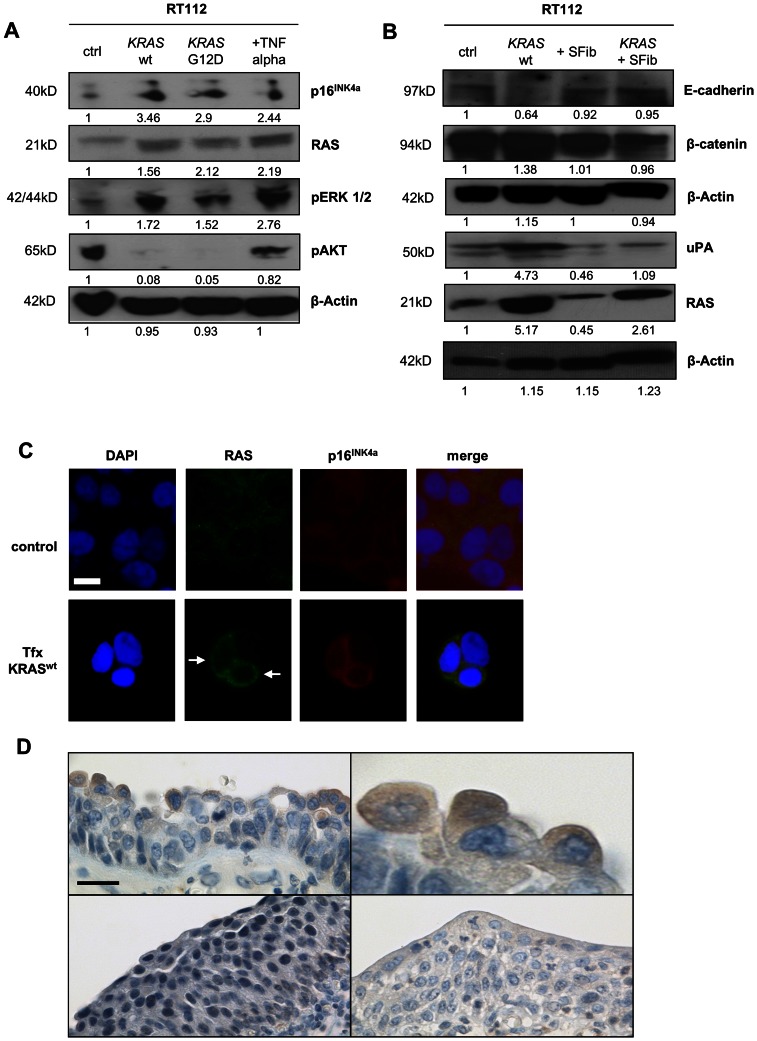
Activation of MAPK signaling leads to overexpression of p16^INK4a^ and uPA. **A**, Western immunoblotting shows an increase of p16^INK4a^, RAS and pERK1/2 upon *KRAS* wt/*KRAS* G12D-transfection and TNFα treatment. Levels of pAKT are elevated in control cells and upon TNFα treatment. **B**, *KRAS* transfection has no effect on the overall Beta-catenin level, but reduces E-cadherin levels and leads to overexpression of urokinase plasminogen activator (uPA). Both effects are reversed upon application of 5 µM Sorafenib (SFib) after transfection. Quantitative gel analysis was done using ImageJ software, v. 1.46r, NIH, Bethesda, USA. **C**, Overexpression of p16^INK4a^ in KRAS-overexpressing RT112 cells upon transfection **(arrows)**. **D**, Expression of uPA in apical tumor cells of urothelial carcinoma in situ (top), but not in healthy or inflamed urothelium (bottom). *Scale bar in Fig. 3C: 10 µm; in Fig. 3D: 50 µm*.

Immunoblotting for p16^INK4a^, RAS and pERK1/2 showed moderate protein levels in untreated RT112 urothelial carcinoma cells, while there was a strong pAKT signal (Figure 3A; complete blots are shown in Figure S4). After transfection with either wild-type or G12D-mutated (constitutively active) *KRAS*, we detected stronger signals for p16^INK4a^, RAS and pERK1/2 while there were no detectable levels of pAKT. Treatment with 20 ng TNFα slightly enhanced the level of p16^INK4a^, while there was strong expression of RAS, pERK1/2 and pAKT. RAS-dependent overexpression of p16^INK4a^ could be confirmed by immunofluorescence microscopy (Figure 3C, lower panel). Taken together, these results confirm upregulation of p16^INK4a^ upon transfection-induced activation of the RAS/MAPK signaling pathway.

Western immunoblotting for E-cadherin and Beta-catenin in *KRAS* wild-type transfected RT112 cells showed a decrease in E-cadherin, while Beta-catenin levels remained unchanged (Figure 3B). Secondly, there was a strong increase in urokinase plasminogen activator (uPA) expression after *KRAS* transfection. Both effects could be reversed by application of the multi-kinase-inhibitor Sorafenib (5 µM). This shows that transfection-induced activation of the RAS/MAPK signaling pathway is sufficient to cause EMT *in vitro*, and that this effect can be inhibited by Sorafenib.

### Expression of urokinase plasminogen activator (uPA) in UCIS

Immunohistochemistry revealed a positive staining pattern for uPA in the cytoplasm of apical tumour cells (Figure 3D, top panel). There was no staining signal in healthy or inflamed urothelium (Figure 3D, bottom panel). This confirms uPA expression in UCIS *in vivo*.

## Discussion

The role of human papillomavirus (HPV) in bladder carcinogenesis remains controversial. In cattle, infection with bovine papillomavirus (BPV) is a central risk factor for development of urothelial cancer[20], [21]. In humans, overexpression of p16^INK4a^ – which is regarded as a surrogate marker for infection with oncogenic HPV in other tumours - has been described for urothelial carcinoma *in situ* (UCIS). Here, we show for the first time that this overexpression of p16^INK4a^ in UCIS is neither associated with oncogenic HPV infection nor p16^INK4a^ gene amplification. Furthermore, we point out a mechanism for p16^INK4a^ upregulation in UCIS.

In slight contrast to the findings by Yin et al.[11], who found strong p16^INK4a^ staining in 100% of examined UCIS, we found nuclear and cytoplasmic positivity for p16^INK4a^ in 92.6% of UCIS. This might be due to the higher sample number of isolated UCIS in our group; however, these findings are comparable to figures from literature[22]. For CIN lesions, all included samples showed positivity for p16^INK4a^, which is also consistent with data from the literature[23]. We detected high-risk HPV (genotype 16) DNA in only 80% of CIN samples. Although p16^INK4a^ is regarded as a surrogate marker for HPV infection in these lesions, this is comparable to findings in literature, where a positive predictive value (PPV) of p16^INK4a^ for high-risk HPV infection between 59 and 91% has been described [24], [25].

Surprisingly, we did not detect HPV DNA in any of the examined UCIS samples. This finding is in contrast to results from Shigehara et al, who found HPV DNA in 18 out of 117 (15%) bladder carcinomas in 2011[26]. 15 (83%) of the HPV-positive tumours in that study displayed a noninvasive growth pattern (that had not been further specified into papillary or flat lesions), and most of the HPV-positive tumours had been classified as low-grade. 17 (94%) of the HPV-positive cases in the study had also shown positive p16^INK4a^ immunostaining. Contrary to our study, that study did not focus on flat urothelial lesions/urothelial carcinoma *in situ*, and accordingly, the same group and others had previously found evidence for an etiologic role of human papillomavirus in papillary and low-grade urothelial lesions[27], [28]. Other authors, however, do not state an association between HPV infection, p16^INK4a^ expression and inverted papillomas of the urinary bladder[29]. The described differences around the world might also be caused by different distribution of risk factors; for example, presence of HPV DNA in bladder cancer might be associated with Schistosomiasis-induced tumourigenesis[30].

Our own results are consistent with data for invasive urothelial carcinoma that is widely regarded to be more closely related to UCIS than papillary lesions in terms of molecular tumourigenesis[31], [32]; for example, Yavuzer et al. and Ben Selma et al. did not find HPV DNA in a total of 195 urothelial carcinomas[33], [34]. Alexander et al.[15] just recently reported about a series of 69 squamous cell carcinomas and urothelial carcinomas with squamous differentiation of the bladder, none of which was positive for HPV DNA. Interestingly, 31 and 33% of the carcinomas in that study had also shown positivity for p16^INK4a^, and upregulation of p16^INK4a^ in tumor cells independent of HPV infection has also been described for other tumour entities[35]. Furthermore, a 2010 study showed that even in HPV DNA-positive bladder cancers, p16^INK4a^ expression did not correlate with the expression of HPV16 E7 oncoprotein[36]. Taking these and our results together, we think that it is not advisable to regard p16^INK4a^ as a surrogate marker for HPV infection or HPV-associated tumorigenesis in urothelial carcinoma. However, the possibility remains that other viral infections contribute to urothelial carcinogenesis. For human polyomavirus (HPyV), however, such an association between infection status and bladder cancer risk could not be shown in a previous study[14].

FISH analysis showed a regular number of the *CDKN2A* gene locus encoding for p16^INK4a^ in most of examined UCIS nuclei. There was no amplification, but loss of one gene copy in about one fifth of nuclei in the cancerous lesion. This finding is consistent with data from the literature, where multiple studies described loss of *CDKN2A* in flat urothelial lesions and urothelial carcinoma[2], [37]. Our second conclusion is therefore that overexpression of p16^INK4a^ is not related to gene amplification in UCIS.

Strong expression of p16^INK4a^ in UCIS was accompanied by enhanced kinase phosphorylation as shown by immunohistochemistry. Since overexpression of p16^INK4a^ upon activation RAS/MAPK signaling has been described[38], we postulated that there might be increased signaling activity of that pathway in UCIS. Accordingly, we found that vector-based overexpression of KRAS *in vitro* enhanced phosphorylation of ERK and led to upregulation of p16^INK4a^. However, contrary to the results from immunohistochemistry, we found that treatment with TNFα to simulate an inflammatory setting enhanced kinase phosphorylation and led to upregulation of p16^INK4a^. Since this *in vitro* effect has been previously described[39], one explanation might be that tissue concentrations of inflammatory messengers *in vivo* are lower than they are in cell culture. Furthermore, *in vitro* models lack anti-inflammatory molecules (such as CXCL9–11 and TNFSF14) that have been shown to be upregulated in bladder samples from patients with ulcerative interstitial cystitis and might have influence on apoptotic signaling cascades and p16^INK4a^ expression *in vivo* [40].

The observed changes in E-cadherin and Beta-catenin expression and distribution are consistent with epithelial-mesenchymal transition (EMT), a process in which epithelial cells lose cohesiveness and gain a mesenchymal phenotype as a prerequisite for subsequent invasion[41]. Interestingly, discohesiveness is a key morphologic feature of UCIS[42]. EMT is linked to MAPK signaling, since activity of that pathway has previously been shown to be required for transforming growth factor beta (TGFβ)-induced EMT and is associated with invasiveness of urothelial carcinoma cells[43], [44]. Moreover, high MAPK activity has been linked to the maintenance of bladder cancer stem cell characteristics[45].

Besides EMT induction, RAS signaling led to overexpression of Urokinase plasminogen activator (uPA), and both effects could be suppressed by application of the multi-kinase inhibitor Sorafenib *in vitro*. Sorafenib has been previously shown to effectively inhibit EMT and is thus an interesting candidate for pharmacologic prevention of invasiveness[46]. UPA, on the other hand, is an important inductor of EMT in breast carcinoma[47]. Interestingly, uPA expression, as well as expression of p16^INK4a^, is controlled by the ETS1/2 transcription factor family downstream the RAS/MAPK signaling pathway[48]. Accordingly, we could show enhanced uPA expression in UCIS cells applying immunohistochemistry.

## Conclusions

**Figure 4:**
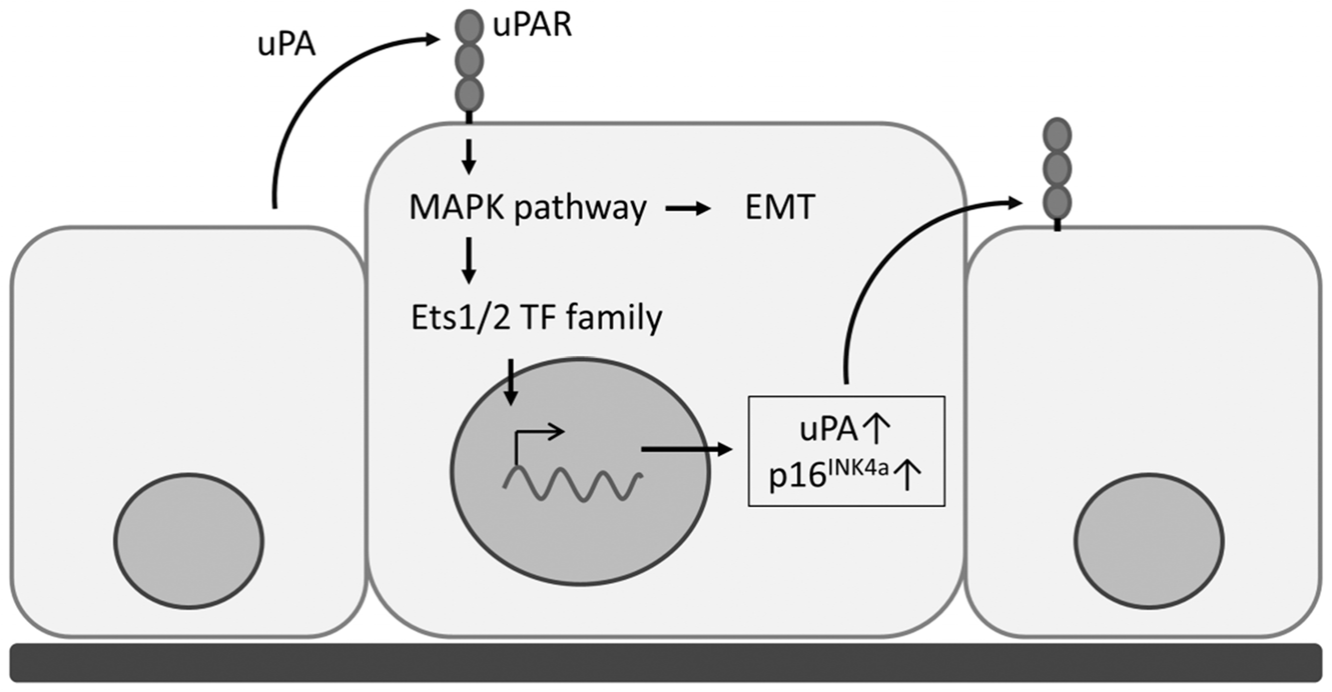
Proposed model for the overexpression of p16^INK4a^ in UCIS. Paracrine activation of uPAR via secreted uPA enhances MAPK signaling and thus initiates EMT in UCIS cells. Besides, MAPK signaling leads to overexpression of p16^INK4a^ and uPA through enhanced ETS1/2 transcription factor activity.

Taken together, we conclude that overexpression of p16^INK4a^ in UCIS is independent of HPV infection and p16^INK4a^ gene amplification, but follows enhanced RAS/MAPK signaling accompanied by upregulation of uPA and epithelial-mesenchymal transition. In our model, which is schematically depicted in Fig. 4, paracrine secretion of uPA enhances MAPK signaling and ETS transcription factor activity, leading to EMT and supporting uPA expression in UCIS cells; overexpression of p16^INK4a^ would therefore be a byproduct of this process and a candidate marker for the EMT which is occurring in the tumour cells. Since in our *in vitro* model, uPA expression as well as EMT are both sensitive to kinase inhibition, our results indicate a potential therapeutic use of Sorafenib to prevent UCIS invasiveness and progression.

## Supporting Information

Figure S1 **A**, Negative immunostaining for p16^INK4a^ in erosive urocystitis. **B**, Negative immunostaining for pAKT in urothelium (above left) and UCIS (below left) compared to moderate staining intensity for pERK1/2 in urothelium (above right) and strong staining intensity in UCIS (below right).
(TIF)

Figure S2 **Authentication of RT112 urothelial carcinoma cells by short tandem repeat (STR) profiling.**
(TIF)

Figure S3 ***KRAS*** **strip assay testing of RT112 cells upon transfection with wild-type** ***KRAS*** **(1),** ***KRAS*** **G12D (2) and testing of untransfected cells** (3). **The band in (2) indicates successful insertion of** ***KRAS*** **G12D mutation.**
(TIF)

Figure S4 **Full-length western blots from the** **Figures 3A and B** **.**
(TIF)

Table S1 **List of antibodies and concentrations as used in the study.**
(DOCX)
