# Supplementary figures and images for: Overexpression of p16INK4a in Urothelial Carcinoma In Situ Is a Marker for MAPK-Mediated Epithelial-Mesenchymal Transition but Is Not Related to Human Papillomavirus Infection
Source: PLoS One. 2013 May 28;8(5):e65189. doi: 10.1371/journal.pone.0065189 (PMC3665800; doi:10.1371/journal.pone.0065189)

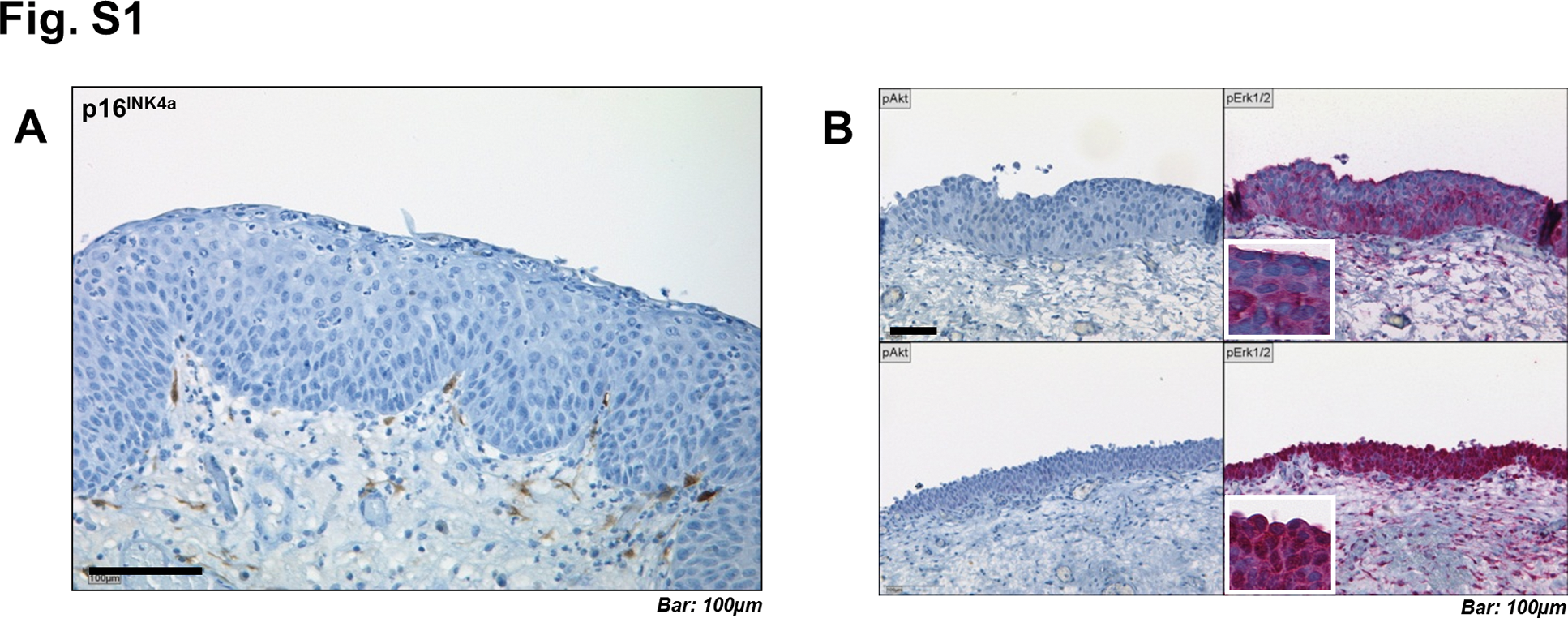

Supplement: Figure S1 — A, Negative immunostaining for p16INK4a in erosive urocystitis. B, Negative immunostaining for pAKT in urothelium (above left) and UCIS (below left) compared to moderate staining intensity for pERK1/2 in urothelium (above right) and strong staining intensity in UCIS (below right). (TIF) [file pone.0065189.s001.tif]

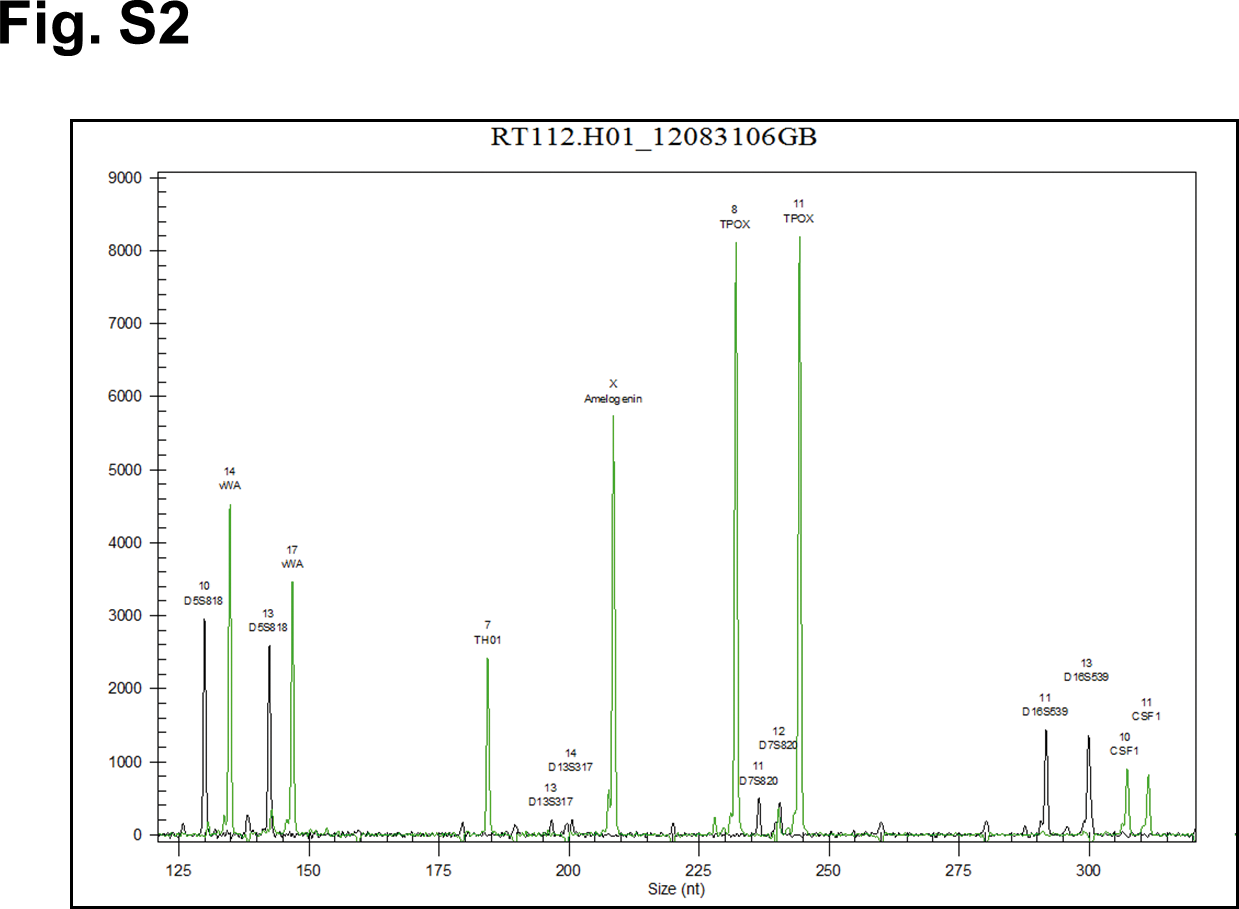

Supplement: Figure S2 — Authentication of RT112 urothelial carcinoma cells by short tandem repeat (STR) profiling. (TIF) [file pone.0065189.s002.tif]

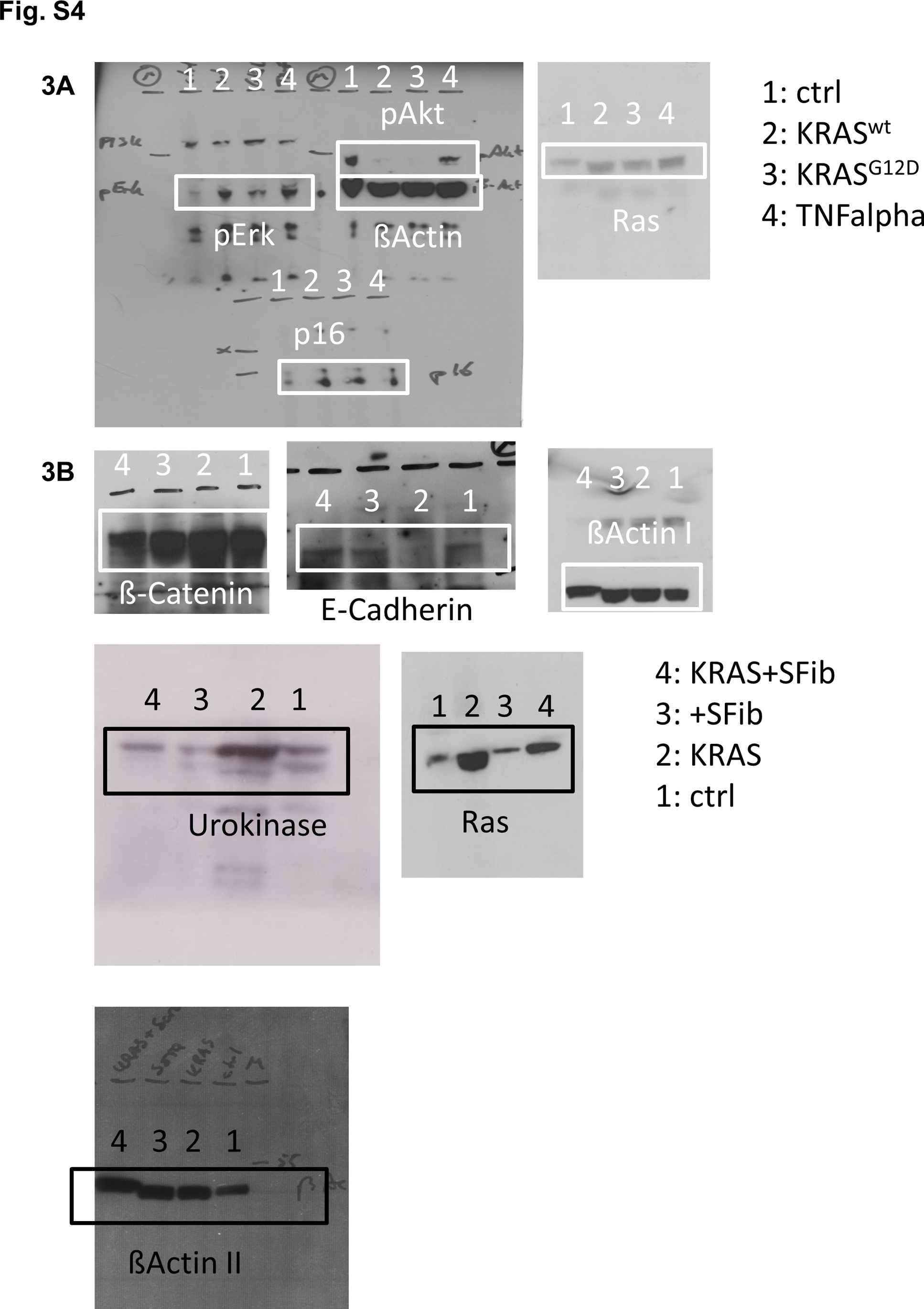

Supplement: Figure S4 — Full-length western blots from the Figures 3A and B . (TIF) [file pone.0065189.s004.tif]
