# Supplementary material for: Overexpression of p16INK4a in Urothelial Carcinoma In Situ Is a Marker for MAPK-Mediated Epithelial-Mesenchymal Transition but Is Not Related to Human Papillomavirus Infection
Source: PLoS One. 2013 May 28;8(5):e65189. doi: 10.1371/journal.pone.0065189 (PMC3665800; doi:10.1371/journal.pone.0065189)
Supplement: Table S1 — List of antibodies and concentrations as used in the study. (DOCX) [file pone.0065189.s005.docx]

**Supplementary Table S1: List of antibodies used in the study**

|  |  | **Conc.** | | |
| --- | --- | --- | --- | --- |
| **Primary Antibodies** | **Supplier** | **IHC** | **IB** | **IF** |
| CINtec® p16 (mouse monoclonal; clone E6H4) | Ventana Medical Systems (Tucson, USA) | prediluted | - | - |
| p16^INK4a^ (mouse monoclonal) | Abcam (Cambridge, UK) | - | 1:500 | 1:250 |
| Ki-67 (rabbit monoclonal; clone 30-9) | Ventana Medical Systems (Tucson, USA) | prediluted | - | - |
| E-cadherin (mouse monoclonal) | Novocastra/Leica, Newcastle Upon Tyne, UK) | - | 1:1000 | - |
| E-cadherin (mouse monoclonal; clone 36) | Ventana Medical Systems (Tucson, USA) | prediluted | - | - |
| Cytokeratin 20 (rabbit monoclonal; clone SP33) | Ventana Medical Systems (Tucson, USA) | prediluted | - | - |
| Beta-catenin (mouse monoclonal; clone 14) | Ventana Medical Systems (Tucson, USA) | prediluted | - | - |
| Phospho-AKT(Ser473) (rabbit monoclonal) | Cell Signaling Tech. (Boston, USA) | 1:200 | 1:1000 | - |
| Phospho-p44/42 MAPK (ERK1/2) (rabbit monoclonal) | Cell Signaling Tech. (Boston, USA) | 1:200 | 1:1000 | - |
| Urokinase plasminogen activator (uPA/PLAU; rabbit polyclonal) | Epitomics (Burlingame, USA) | 1:100 | 1:200 | - |
| Pan-RAS (mouse monoclonal) | Abcam (Cambridge, UK) | - | 1:1000 | 1:1000 |
| β-Actin (mouse monoclonal) | Abcam (Cambridge, UK) | - | 1:10000 | - |
| **Secondary Antibodies** |  |  |  |  |
| Goat polyclonal anti-rabbit IgG - H&L (HRP-conjugated) | Abcam (Cambridge, UK) | - | 1:5000 | - |
| Rabbit polyclonal anti-mouse IgG - H&L (HRP-conjugated) | Abcam (Cambridge, UK) | - | 1:10000 | - |
| Goat polyclonal anti-mouse (Texas Red^TM^- conjugated) | Abcam (Cambridge, UK) | - | - | 1:1000 |
| Goat polyclonal anti-rabbit (DyLight®488- conjugated) | Vector Laboratories (Burlingame, USA) | - | - | 1:1000 |

***IHC, immunohistochemistry; IB; immunoblotting; IF; immunofluorescence.***
